# The Temporomandibular Joint Compromise (TMJC) as a Cause of Otoneurological Symptoms: Headaches, Dizziness and Tinnitus

**DOI:** 10.1055/s-0046-1819593

**Published:** 2026-05-05

**Authors:** Rafael Casañas, Isabel González-Esmorís, Jose Cabrera, Víctor Pérez-Candela, Pedro Saavedra, José Larena-Avellaneda

**Affiliations:** 1 Department of Ear, Nose, and Throat (ENT), Hospital Vithas Santa Catalina, Las Palmas de Gran Canaria, Las Palmas, Spain; 2 Department of Odontology, Private Odontology Practice, Betanzos, La Coruña, Spain; 3 Department of Neurology, Complejo Hospitalario Universitario Insular-Materno Infantil, Las Palmas de Gran Canaria, Las Palmas, Spain; 4 Department of Radiology, Hospitales Universitarios San Roque, Las Palmas de Gran Canaria, Las Palmas, Spain; 5 Department of Mathematics, Universidad de Las Palmas de Gran Canaria (ULPGC), Las Palmas de Gran Canaria, Las Palmas,, Spain; 6 Department of Odontology, Private Odontology Practice, Las Palmas de Gran Canaria, Las Palmas, Spain

**Keywords:** Costen syndrome, migraine, dizziness, tinnitus, temporomandibular disorder, temporomandibular joint dysfunction syndrome

## Abstract

**Introduction:**

The prevalence of temporomandibular disorders in the population with chronic or recurrent headaches is too high for a relationship not to exist. Publications propose the examination of the masticatory system in all patients with headache.

**Objective:**

To introduce a new entity within temporomandibular disorders, temporomandibular joint compromise (TMJC), mandibular movement limiting pathology of extra-articular traumatic cause, and evaluate the response rate and safety of treatment. The limiting cause of mandibular movement is the lack of space between the mandibular ramus and maxilla. The main symptoms of TMJC include headache, dizziness, and tinnitus. In many cases, the headache is previously diagnosed as a migraine.

**Methods:**

Data were collected from 54 patients aged between 6 and 59 years who had a confirmed diagnosis of migraine (according to the the International Classification of Headache Disorders, 3rd edition [ICHD3]) and were treated for TMJC in a dental clinic. They also presented other otolaryngological symptoms. A retrospective quasi-experimental study without a control group was carried out due to ethical considerations related to the harmless nature of the treatment.

**Results:**

After treatment of TMJC, migraine symptoms disappeared in 52 patients (96.3%, *p* < 0.001) and persisted, although with clinical improvement, in 2 (3.7%). There was also an improvement in the other associated symptoms: dizziness disappeared in 23 out of the 27 affected patients (85.21%, *p* < 0.001) and tinnitus disappeared in the 31 affected patients (100%, *p* < 0.001). No significant treatment-related side effects were observed.

**Conclusion:**

The present study shows the high rate of response and safety of the treatment of TMJC.

## Introduction

The aim of the present article is to introduce a new entity, temporomandibular joint compromise (TMJC), which causes headaches and other otorhinolaryngological symptoms, among which dizziness and tinnitus stand out.

In 1934, Costen 1 linked temporomandibular joint (TMJ) dysfunction with headache and other ear, nose, and throat (ENT) symptoms, such as “stuffy” sensation in ears, tinnitus, otalgia, and dizziness, in a group of 11 patients with overbite (the upper incisors overlap the lower incisors). When the overbite was corrected with the appropriate dental prosthesis, the ENT symptoms and headache disappeared. Costen located the origin of the symptoms in the TMJ, which, due to the overbite, exerts pressure on the auriculotemporal nerve and causes irritation of the middle fossa meninge due to its anatomical proximity to these structures. These causal theories have already been discarded. 2

What Costen described eventually became known as Costen's syndrome (a pathology of the joint that Dr. Costen never explicitly mentioned) and is now better known as craniomandibular dysfunction (CMD). Since 1993, the American Academy of Orofacial Pain (AAOP) has used the generic term *temporomandibular disorder* , which covers a wide variety of clinical problems of the TMJ, the masticatory muscles and their associated structures. These dysfunctions are the most frequent cause of non-dental pain in the orofacial region and are a growing problem in recent years. 3 The temporomandibular disorders of the AAOP were later incorporated into category 11 of the International Classification of Headache Disorders, 3rd edition (ICHD-3). 4

**Fig. 1:**
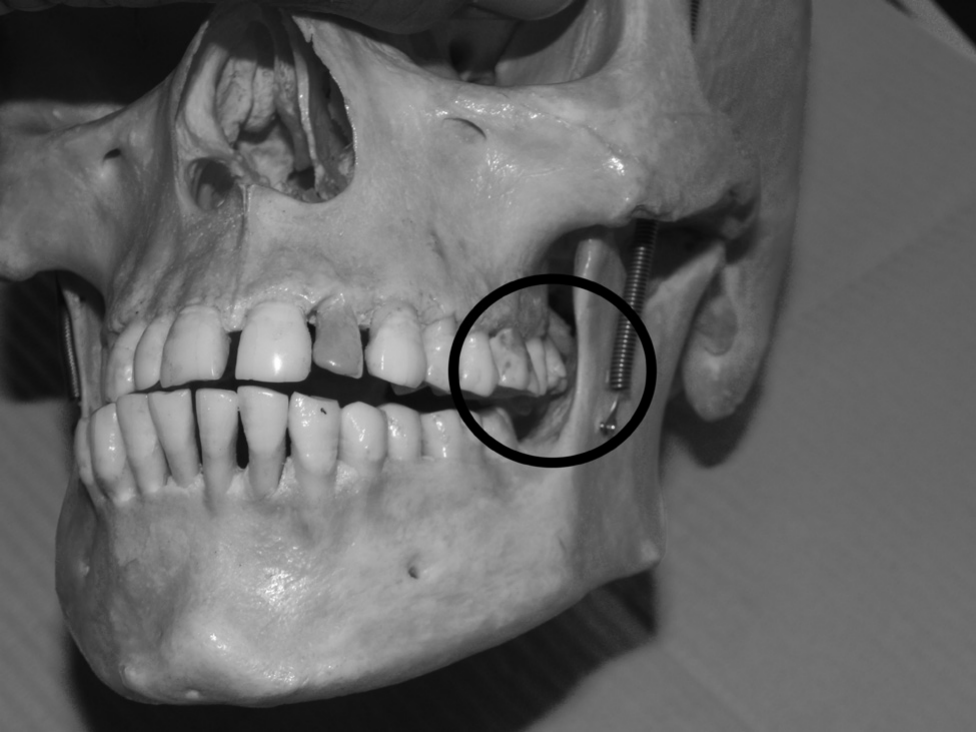
Image of right mandibular laterality movement compromised by the lack of space on the left side. It shows how the range of movement is limited by the impact of the internal face of the left mandibular ramus against the extruded left upper molar, generating temporomandibular joint compromise.

Our intention is to describe TMJC as a new entity within this group of temporomandibular disorders. Temporomandibular joint compromise is not a joint disorder, it is a traumatic pathology that limits mandible movement due to the impact of the mandibular ramus against the maxilla, caused by the lack of space between these two structures. The inclusion of the term “compromise” in the name of this entity is based on the fact that a joint is considered “compromised” when its range of motion is restricted by injury or disease. The limiting cause of the movement in the TMJC is the lack of space between the mandibular ramus and the maxilla, which causes the impact of some structure located in the mobile part of the masticatory apparatus against the posterior area of the maxilla ( Fig. 1 ). Common causes that reduce this space are bone exostoses, extruded teeth, oversized dental prosthesis, compression due to rest or chewing habits and increased divergence of dental arches due to orthodontics. These situations limit the free movement of the mandible and its muscles during chewing, swallowing, phonation or yawning.

Temporomandibular joint compromise was first described by Larena-Avellaneda. 5 6 This author observed the resolution of multiple comorbidities in a patient after the resolution of a disorder at the posterior level of the maxilla, with no other explanation found.

The main symptoms of TMJC are acute and chronic headaches, dizziness and tinnitus, with less common symptoms including burning mouth syndrome, hyperacusis, and xerophthalmia. In many cases, the headache is clinically compatible with migraine and patients have been previously diagnosed with migraine. Temporomandibular joint compromise symptoms are clearly different from those of CMD (joint pain, snapping, and functional impotence) and have been widely associated with temporomandibular disorders in the literature:

- *Migraine* is highly prevalent, affecting all age groups. There is no curative treatment at present, and recurrences can happen throughout life. Migraine is now a major public health problem with a high economic impact. It is found to be the 3rd highest cause worldwide of years lost due to disability in 2021 according to the World Health Organization. 7 The exact pathogenic mechanism of migraine is unknown, but the trigeminovascular system has been described as responsible for the pain. 8 This system would be activated secondary to a trigeminal discharge. In chronic craniofacial pain, there is prior sensitization, both peripherally and centrally generated by a chronic or repeated stimuli, that can promote chronic pain conditions. 9 10 11 The current paper hypothesizes that TMJC, by compressing the tissues and structures between the mandibular ramus and the maxilla, is the trigger of that trigeminal discharge, thus activating the necessary sequence: trigeminal discharge > trigeminovascular system sensitization > migraine. Other authors have also suggested the role of masticatory dysfunction in the pathogenesis of headache and migraine, 11 and there are many bibliographical references that have identified a high association between migraine and temporomandibular disorders, 12 13 finding temporomandibular disorders in 78% of patients with episodic migraine and in 100% of patients with chronic migraine. 14
- The same goes for patients experiencing *dizziness* . Many studies find an epidemiological relationship between migraine and vertigo 15 probably because they share physiopathological principles. Published series report a migraine incidence of 35% in patients with vertigo. 16 When migraine is associated with vertigo (25–50% of migraine episodes 17 ), it can have both peripheral and central characteristics. 18 Based on these data, *vestibular migraine* is recognized as an independent entity in ICHD-3. 19 Vestibular migraine is an increasingly well-known but still under-diagnosed entity, 20 and may be the most frequent entity of episodic vertigo. 21 It is also suspected that migraine shares physiopathological principles with benign paroxysmal vertigo in childhood, 22 an entity also included in ICHD-3 and considered a possible migraine precursor. Also, patients with Meniere's disease and benign paroxysmal positional vertigo have a high prevalence of migraine. 16 17 19 20 23 24 25 The current paper hypothesizes that the trigeminal discharge induced by TMJC alters the correct functioning of the reticular system in the brainstem, thereby altering the functioning of the vestibular system.
- *Tinnitus* is another common TMJC symptom. Only in 5 to 10% of the patients with tinnitus a cause is found. 26 Tinnitus is eight times more prevalent in patients with temporomandibular disorders. 2 When both situations are associated (tinnitus and temporomandibular disorder), it mainly affects young people. 27 Patients often explain how extreme movements of the mandible exert a “liberating” effect on their tinnitus or how the tinnitus changes intensity with chewing, and no direct relationship for this has been found to date. Clonic contractures of the muscles of the palate, Eustachian tube, malleus or stapes may produce tinnitus 26 and simulate Eustachian-tube dysfunction. 28 29

The aim of the present study is to determine the response rate and safety of TMJC treatment in patients with migraine and other ENT symptoms.

## Methods

### Diagnosis of TMJC

**Fig. 2:**
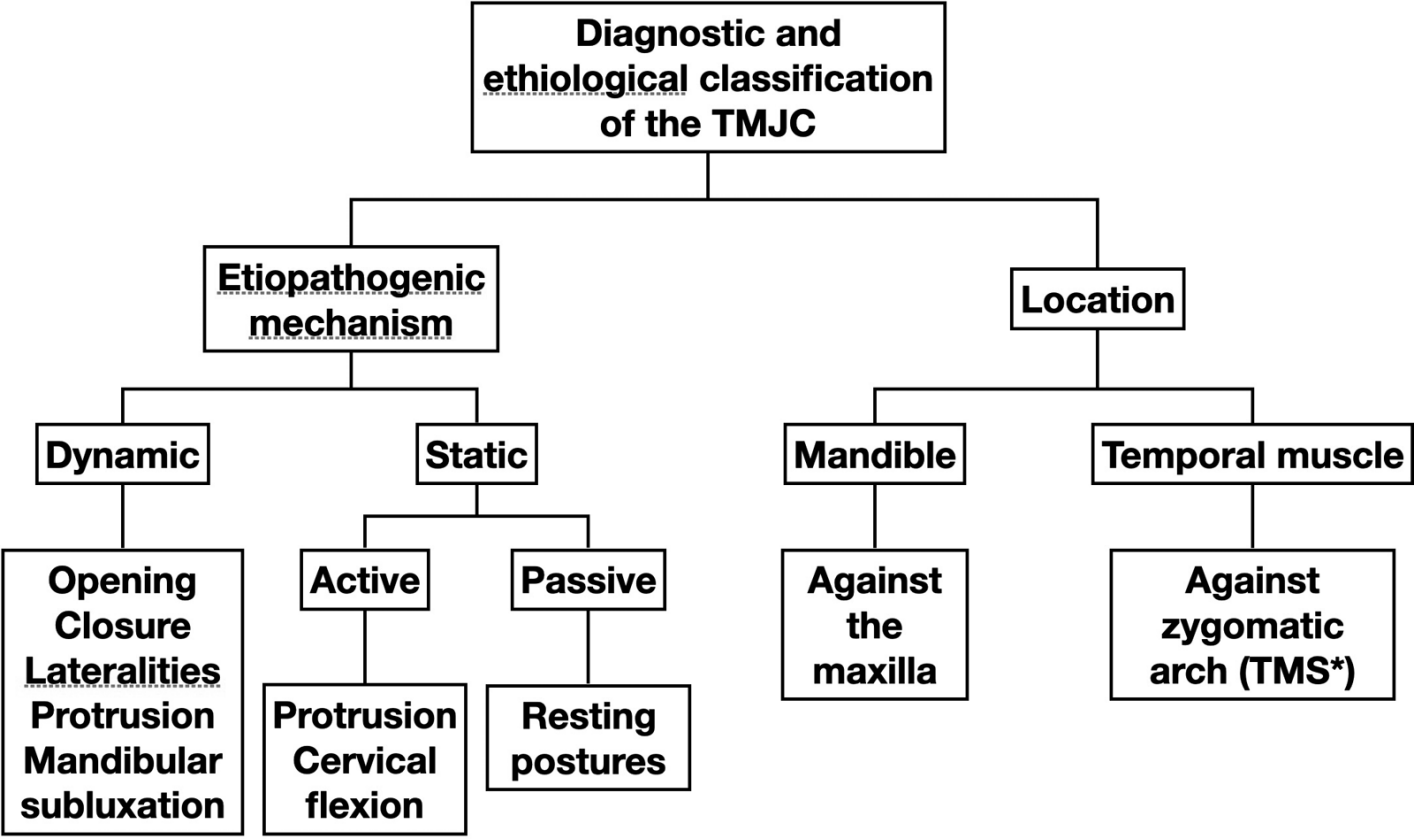
Classification of temporomandibular joint compromise according to the etiopathogenic mechanism and its location. *Temporal muscle syndrome.

Clinical history was the main tool for diagnosing TMJC. It is fundamental to analyze the chronology of the pain onset to identify the etiopathogenic mechanisms of TMJC, both static or dynamic, so that they can be classified ( Fig. 2 ) in order to apply the necessary treatment guidelines to eliminate these causal mechanisms.

**Fig. 3:**
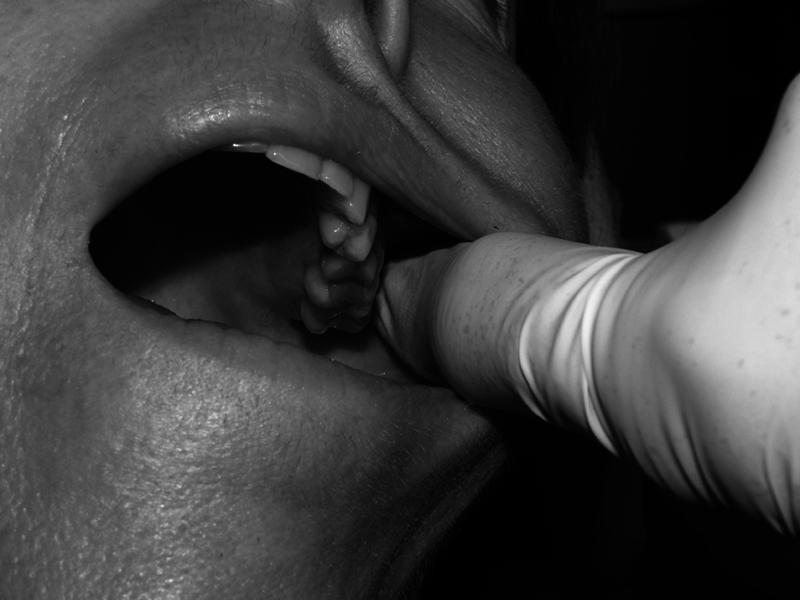
Palpation of the left jugal area on the inner side of the mandibular ramus, referred to as the “temporomandibular joint compromise area”.

First, ear, nose, and throat disease was ruled out as a cause of chronic pain, and a functional and neurological examination of the masticatory apparatus was performed. The extraoral functional exploration started with palpation of the TMJs and the coronoid processes, requesting the patient to open and close the mouth slowly to identify if there was pain (“positive palpation” was noted if pain was present), and also assessing whether it was unilateral or bilateral. The presence or absence of subluxation of the TMJs was checked. In the intraoral functional examination, it was evaluated if there was sufficient free space between the mandibular ramus and the maxilla, which we refer to as the “TMJC area” ( Fig. 3 ), during opening, closing, and lateral movements of the mandible. Intraoral palpation was used to locate the painful area of the TMJC on each side (“positive palpation” was noted if pain was present).

Special attention was given to the intraoral functional examination of the dental closure and to any mandibular deviation due to occlusal interference, as this forces the mandible into an advanced position. The detailed analysis of the direction and magnitude of the mandibular deviation was important because it marked the approach for selective carving. We also explored mandibular laterality movements to find out the usual chewing side, a habit that must be taken into account when setting treatment guidelines.

Neurological examination included assessment of cranial nerves, corneal, nasal, and nausea reflexes, along with evaluation of tactile and painful sensitivity in the facial skin, oral mucosa, and tongue. Reflexes were assessed as normal or decreased, and sensitivities were assessed as normal or decreased (hypoesthesia and hypoalgesia).

Efforts were made to determine the “higher TMJC side”. Temporomandibular joint compromise is usually bilateral, but there is always a more symptomatic side, which is called the “higher TMJC side”.

Orthopantomography was used as a routine complementary test. This image identifies the included upper molars and the size of the tuberosities to calculate the free space that could be achieved after their removal with surgery.

### TMJC Treatment

**Fig. 4:**
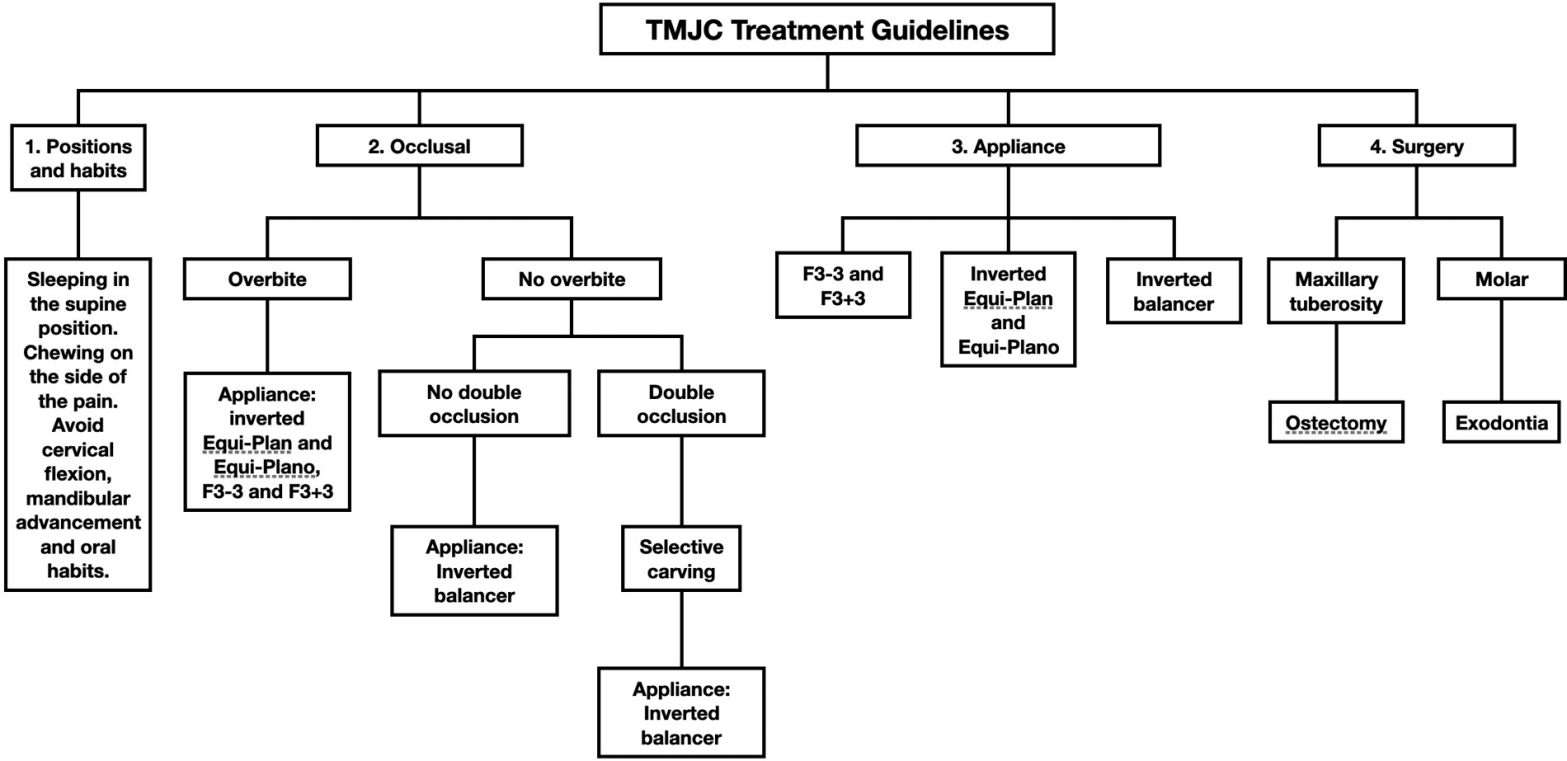
Temporomandibular joint compromise treatment guidelines.

Once the TMJC was identified and classified, the treatment strategy was based on four guidelines: 1 - *positions and habits* ; 2 - *occlusal* ; 3 - *appliances* ; 4 - *surgical* ( Fig. 4 ). Temporomandibular joint compromise treatment is mainly dental, but, as we will see below under *technical information* , the first guideline, *positions and habits* , is the most important for doctors in our clinics because it includes the recommendations that we can give to patients before referring them to a specialized dental clinic. Many patients will present a significant improvement only with these recommendations. For the other 3 guidelines, we will need a specialized dentist.

#### Positions and Habits Guidelines

First, the patient will be advised on the sleeping posture and the side on which to chew. Additionally, we advise them to stop performing certain oral habits (eating pipes, chewing gum, onychophagy…), activities (sewing, computer work, text messaging…) or sports (swimming, weightlifting, cycling…) that involve flexing the neck forward or advancing the mandible.

#### Occlusal Guideline

In this phase of the treatment, the dental contact surface is adjusted to eliminate occlusal interferences in the closing path and in the lateral movements through selective carving systematized by Larena-Avellaneda. 30

#### Appliance Guideline

A new appliance designed by Larena-Avellaneda and González-Esmorís 31 32 33 34 35 was used, associated with other devices based on Neuro-Occlusal Rehabilitation 36 modified for the treatment of TMJC.

#### Surgery Guideline

Extraction of last upper molar on each side and ostectomy by surgical milling of bone tuberosities of the maxilla.

### Selection and Description of Participants

The present quasi-experimental retrospective study was planned in June 2014 on a cohort of 325 patients who had been treated for TMJC in a dental clinic between June 1998 and May 2014.

From the initial sample, only patients meeting the following criteria were included:

1. Diagnosis of migraine confirmed by a neurologist according to the 2018 ICHD-3 criteria.
2. Fulfillment of the treatment indicated for TMJC.
3. Agreement to conduct a monitoring visit between July and December 2014.

The final sample consisted of 54 patients between 6 and 59 years of age ( Table 1 ).

**Table 1:** Numerical variables are presented as means and standard deviations (SDs) or medians and interquartile ranges (IQRs)

Number of participants		54
**Age, years**		28.3 (14.5–38.2)
	≤ 14 years	13 (24.1)
	14–18 years	3 (5.6)
	> 18 years	38 (70.4)
**Male/Female**		
	Female	48 (88.9)
	Male	6 (11.1)
**Follow-up time, months**		27.3 (10–60.9)
**Reason for consultation**		
	Not migraine	12 (22.2)
	Right migraine	12 (22.2)
	Left migraine	14 (25.9)
	Bilateral migraine	16 (29.6)
**Initial diagnosis of migraine**		
	Neurologist	23 (42.6)
	Others	25 (46.3)
**Routine pain medication**		47 (87.0)
**Orthodontic treatment**		11 (20.4)
**Discharge splints**		19 (35.2)
**Nocturnal migraine chronology**		
	Wakes up the patient	25 (46.3)
	Dawns with the pain	33 (61.1)
**Exploratory findings**		
	Change of chewing side	16 (29.6)
	Double occlusion	34 (63.0)
**Characteristics of the intervention**		
	Postures and habits	54 (100.0)
	Occlusal	31 (57.4)
	Appliances	51 (94.4)
	Surgery	25 (46.3)

**Note** : Categorical variables are presented as frequencies and percentages.

Both at the start of treatment and at the control visit, the same examinations were carried out.

The extended time interval permitted the inclusion of both very old and very new patients, resulting in a follow-up period with a median of 27.3 months.

The patients included in the study or their legal guardians received the necessary information and provided prior informed consent. The study was certified by a hospital ethics committee. This committee approved not to include a control group due to ethical considerations related to the harmless nature of the treatment.

### Technical Information

The *positions and habits guideline* was applied to 100% of the patients. First, the position of sleeping or resting is evaluated. If the pain wakes the patient during the early morning (46.3%) or if the patient has pain upon waking up in the morning (61.1%), it is attributed to the resting posture. This is because in the lateral or prone sleeping posture is when etiopathogeny by pillowing occurs. 37 The pain side and the sleeping side are the same because the ipsilateral TMJC area is compressed. Until now, no explanation had been found as to why patients wake up in pain.

If the pain appears throughout the day, the daily activities are analyzed: work postures, physical exercise, hobbies (reading, sewing, text messaging...). Any habit, hobby, or sport that requires neck flexion involves mandible protrusion and the possibility of hitting the TMJC area and is, therefore, contraindicated for patients. This also explains why pain appears after these activities.

If the pain appears or increases after meals, it is related to chewing, and the pain affects the side opposite to the chewing side because unilateral chewing, for example, on the right side, causes the mandible to move to the right, compressing the left TMJC area ( Fig. 1 ). This generates left TMJC, and we must reverse this situation by chewing on the “bad” side, the left, in this case. A soft diet is recommended and chewing on the side that is most painful. Chewing on the side of greater pain may seem wrong, but it is the correct approach in TMJC.

In summary, to prevent compression of the TMJC zone, we recommend the following two things to patients: sleeping on their backs and avoid chewing, but if they want to chew or sleep on one side, we give the next warnings. When chewing, we recommend soft diet using only the painful side to open the affected TMJC zone. In case of sleeping on one side, it has to be the contralateral side to achieve the same effect (open the painful TMJC zone). For example, if the left side is the painful side, we recommend chewing on the left and sleeping on the right.

The *occlusal guideline* (it was applied in 57.4%) depends on the presence or absence of overbite. If there is no overbite, selective carving is necessary if there is double occlusion (63%) to eliminate the occlusal interferences causing the mandible to advance upon closure. It is essential to remove the double occlusion before placing the appliance (next guideline) because otherwise the patient may feel uncomfortable and that causes tension to the appliance, which can lead to its breakage. Occlusal carving is indicated for temporary teeth and permanent teeth above the age of 18.

If there is an overbite, the occlusal guideline is not necessary but if it is applied, it ensures that the appliance will be centrally articulated to avoid tension and to achieve patient comfort in order to facilitate the treatment.

In the *appliance guideline* (applied in 94.4%), it is also the presence or absence of overbite that determines the type of appliance. The TMJC appliance is inverted to contribute to the limitation of mandibular protrusion.

The *surgical guideline* (applied in 46.3%) is performed to extract upper third molars or remove large maxillary tuberosities. The goal of this guideline is to gain space for the mandible to move freely.

### Statistics

The main variable in the current study was the presence or absence of migraine at the control visit. According to the ICHD-3 criteria, the absence of migraine is considered when the patient reports no attacks with characteristics of migraine in the last 3 months. In our study, pain intensity was assessed at the beginning of treatment and at the control visit using a Visual Analog Scale.

Among other secondary variables, we analyzed the recovery of neurological symptoms and signs, such as reflexes, dizziness/vertigo, and tinnitus. The presence or absence of other comorbidities was also assessed: CMD, burning mouth syndrome, or anxiety. The results at the follow-up were compared with the baseline data collected before the start of treatment.

Categorical variables are presented as frequencies and percentages. Numerical variables, such as age and follow-up time, are presented as means and standard deviations (SDs) or as medians and interquartile range (IQR) depending on whether or not the assumptions of normality were made. The medians were compared with the Wilcoxon test for paired data, and the percentages with the McNemar test. A contrast of hypotheses was considered statistically significant when the corresponding *p* -value was < 0.05. The data was analyzed with the R package (R Development Core Team), version 3.1.0.

## Results

At the start of treatment, 33 patients (61.1%) had migraine without aura, and 21 (38.9%) migraine with aura. At the control visit, migraine had disappeared in 52 patients (96.3%, *p* < 0.001), although in 5 of them a headache persisted that no longer met the clinical criteria for migraine. The 2 patients out of the total (3.7%) who were still suffering from migraine with aura reported improvement in both frequency and intensity.

The status of comorbidities and other manifestations at the baseline and at the control visit is shown in Table 2 . In the ENT field, the disappearance of dizziness and tinnitus must be highlighted. Other neurological signs and symptoms disappeared by the time of the control visit ( Table 3 ).

**Table 2:** Incidence of comorbidities and other clinical manifestations before starting treatment and at follow-up

		Baseline	In control	*p* -value
		N = 54	N = 54	
**Migraine**				< 0.001
	No	0	52 (96.3)	
	Without aura	33 (61.1)	0	
	With aura	21 (38.9)	2 (3.7)	
**Comorbidities**				
	Craniomandibular dysfunction	7 (13.0)	0	0.023
	Burning mouth syndrome	4 (7.4)	0	0.134
	Anxiety	10 (18.5)	1 (1.9)	0.008
	Convulsions	4 (7.4)	0	0.134
**Clinical manifestations**				
	Right cranial paresthesias	10 (18.5)	0	0.004
	Left cranial paresthesias	12 (22.6)	2 (3.7)	0.004
	Right xerophthalmia	9 (16.7)	1 (1.9)	0.013
	Left xerophthalmia	10 (18.5)	2 (3.7)	0.013
	Bruxism	25 (46.3)	2 (3.7)	< 0.001
	Right TMJ subluxation	27 (50.0)	4 (7.4)	< 0.001
	Left TMJ subluxation	27 (50.0)	6 (11.1)	< 0.001
	Dizziness	27 (50.0)	4 (7.4)	< 0.001
	Vertigo	12 (22.2)	1 (1.9)	0.003
	Right tinnitus	14 (25.9)	0	< 0.001
	Left tinnitus	17 (31.5)	0	< 0.001
	Meniere's Syndrome	2 (3.7)	0	0.480

**Table 3:** Neurological examination of patients before starting treatment and at follow-up

	Baseline	In control	*p* -value
Diminished right corneal reflex	9 (16.7)	0	0.008
Diminished left corneal reflex	11 (20.4)	0	0.003
Diminished right nasal sneeze reflex	4 (7.4)	0	0.134
Diminished left nasal sneeze reflex	4 (7.4)	0	0.134
Diminished pharyngeal reflex	6 (11.1)	0	0.041
1st right trigeminal branch hypoesthesia	3 (5.6)	0	0.248
1st left trigeminal branch hypoesthesia	7 (13.0)	0	0.023
2nd right trigeminal branch hypoesthesia	5 (9.3)	0	0.074
2nd left trigeminal branch hypoesthesia	8 (14.8)	0	0.013
3rd right trigeminal branch hypoesthesia	4 (7.4)	0	0.134
3rd branch left trigeminal hypoesthesia	7 (13.0)	0	0.023
1st right trigeminal branch hypoalgesia	1 (1.9)	0	1
1st left trigeminal branch hypoalgesia	7 (13.0)	0	0.023
2nd right trigeminal branch hypoalgesia	5 (9.3)	0	0.074
2nd branch left trigeminal hypoalgesia	8 (14.8)	0	0.013
3rd right trigeminal branch hypoalgesia	3 (5.6)	0	0.248
3rd branch left trigeminal hypoalgesia	8 (14.8)	0	0.013

No side effects directly attributable to the treatment itself were observed.

## Discussion

In the present quasi-experimental retrospective study, the aim is confirmed and the result is valid and reliable.

According to the International Headache Society (IHS) guide for the study of preventive treatments for chronic migraine in adults, a reduction of at least 50% in the number of migraine days or the number of moderate or severe headache days has traditionally been defined. 38 The treatment of TMJC resulted in the absence of migraine in 96.3% ( *p* < 0.001) of the studied sample and a clinically significant improvement in the remaining 3.7%. In addition, 23 out of 27 affected patients experienced the disappearance of dizziness (85.21%, *p* < 0.001) and tinnitus in the 31 affected patients (100%, *p* < 0.001). These data indicate strong scientific evidence.

The study evaluates the results at the beginning and at the follow-up visit of this new treatment. Because it is a novel treatment, it is not possible to make a comparison with any previous studies as there is no history of this type of treatment.

The explanation for why the TMJC produces headache is that the impact on the “TMJC area” produces the compression of soft tissues (muscles, connective tissue, vessels, nerves, fascias, oral mucosa, periosteum, etc.) and the stimulation of the trigeminal sensory endings, as well as the sensory and motor fibers of the autonomic system, generating the necessary trigeminal discharge for the activation of the trigeminovascular system. The TMJC activates nociceptive receptors of first-order pseudo-unipolar trigeminal neurons (semilunar ganglion) in its mandibular and maxillary division, especially with the afferences of the buccal nerve, the sensitive branch of the anterior division of V3. The relationships with the autonomic system are established through the connections of the reticular system in the brainstem involving the four parasympathetic nuclei (Edinger-Westphal, superior salivatory, inferior salivatory, and dorsal of the vagus) and/or through irritation of the trigeminal nerve by involvement of the four parasympathetic ganglia (ciliary, sphenopalatine, otic, and submaxillary). All this triggers an eruption of symptoms caused by the connections with the cranial nerves, mainly migraine-like headache, but also instability, dizziness, vertigo, and tinnitus.

The inclusion of a heterogeneous population in the current study allows us to observe that the prevalence of the TMJC affects without distinction of age. A high percentage of patients under 14 years of age was observed (24.1%), probably due to the fact that pediatric neurologists and primary care pediatricians have started, since 2011, to incorporate TMJC in the Protocol for Management of Headache in Pediatric Primary Care 39 in the province of Las Palmas. The high prevalence of TMJC among the pediatric population may be caused by the increased volume generated by the formation and eruption of permanent upper molars at ages 6 and 12, ages that match the childhood migraine peaks. It has also been observed that in order to relieve the pain caused by the eruption of permanent molars, young people acquire the habit of protruding the mandible in order to bite down and compress the mucous membrane swollen by the eruption of those molars. This habit of mandibular advancement leads to TMJC.

**Fig. 5:**
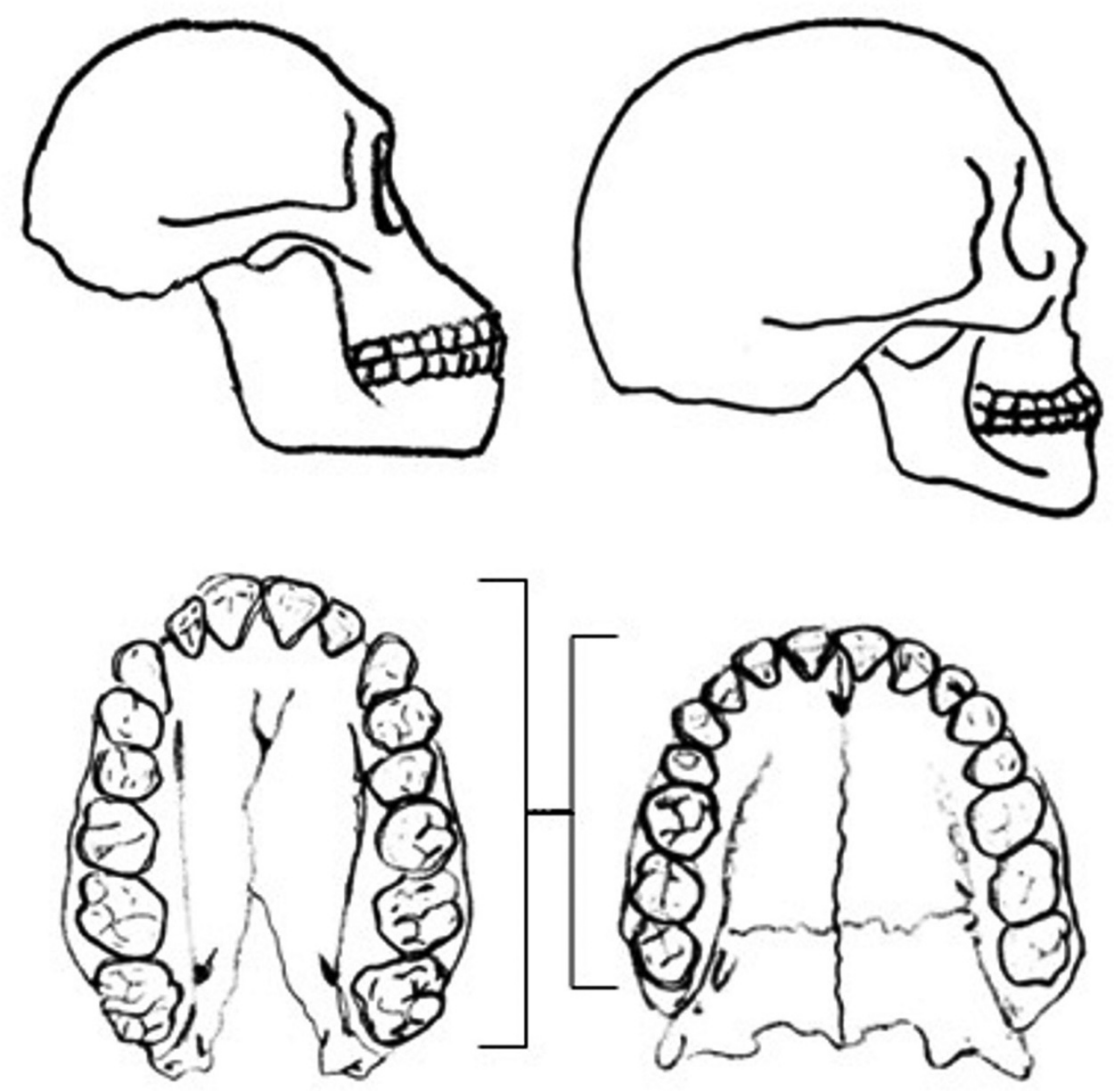
Evolution of the masticatory system. Schematic representation of a skull of Australopithecus (left) and homo sapiens (right). The loss of posterior-anterior development in homo sapiens has led to the widening of the dental arcade to accommodate the same number of teeth.

The higher incidence of TMJC in female patients (88.9%) is due to their lower masticatory effort compared to that of males, resulting in less development and volume of the masticatory apparatus. This is important because TMJC is essentially a “lack of space” issue since volume has been lost in the human masticatory system as a direct consequence of evolution by the “civilized” diet. Our mouth is shorter than that of our ancestors, yet the number of teeth has remained constant ( Fig. 5 ). By reducing the posterior-anterior development and having less muzzle, our dental arches have had to widen at the back to accommodate the same number of teeth, thus stealing space for free lateral movement of the mandible. 40

There are everyday situations that are linked to the etiopathogenic mechanism of the TMJC. Among them, we highlight the *previous* *orthodontic treatments* (20.4% had worn it), which widen the dental arches to align the teeth, and the *occlusal splints* (35.2% were using it), which steal space between mandible and maxilla. This is why orthodontic treatment and occlusal splints are generally contraindicated, as they can exacerbate or cause TMJC. We also see loss of space in TMJC patients with excessively large dentures. These patients improve in a short time by trimming the prosthesis.

The treatment must be done bilaterally because our experience indicates that both sides are usually affected, although the patient refers pain primarily on one side. Symptoms on the major TMJC side may mask the symptoms of the contralateral TMJC. If only the worst side is treated, patients in remission may experience contralateral worsening by no longer masking, mistakenly appearing to be a failure or side effect of the treatment.

There are several issues that highlight the importance of the current study:

- The first, and for us the most significant, is that we achieve the absence of migraine without pharmacological treatment. It is important to emphasize the fact that no new treatment for migraine was tested in the present study. We are treating TMJC, and, for that, we are using novel techniques in stomatology/odontology. The TMJC acts as a trigger for headache and other symptoms that are difficult to control in our daily practice (dizziness, tinnitus, burning mouth syndrome, etc.).
- The second point is the health status of the patients: other serious neurological signs, comorbidities, and significant mood deterioration such as anxiety, coexisted with migraine (18.5%). Most had completed the “healthcare network” available for patients in this situation (neurologists, psychiatrists, otolaryngologists, maxillofacial surgeons, traumatologists, rheumatologists, physiotherapists, acupuncturists, etc.) and the usual radiological tests in these cases had been performed (computed tomography [CT] and magnetic resonance imaging [MRI] of the skull, sinuses, etc.).
- Thirdly, we highlight the rapid resolution of migraine symptoms, in some cases after only 3 months from the start of treatment, and the absence of recurrence after an average of 27.3 months of follow-up. The average time of resolution of TMJC has not been studied, but it is expected to be at least 12 to 18 months, which we estimate to be the minimum amount of time necessary for consolidation of the functional changes of the postural and oral habits, and consolidation of the osseous and dental morphological modifications made.

The present work has various methodological considerations and limitations:

- First, as this was a quasi-experimental retrospective study carried out entirely with patients in a dental clinic, the available resources were limited. This limitation also means that we do not know the prevalence of TMJC in the whole migraine population and in the general population.
- Secondly, if the patient indicated the absence of migraine at the follow-up visit, this was confirmed by reviewing the anamnesis in the medical history. Thus, we avoided overestimating the results due to the so-called courtesy bias, in which the patient tries to please the researcher with favorable responses.
- Third, due to the quasi-experimental retrospective design of the study, it was not possible to establish a temporal exposure-effect association. Nevertheless, it speaks in our favor that a chronic disease like migraine begins to improve very soon after starting treatment for TMJC, reducing the possibility of external factors influencing the results. Furthermore, these chronic patients are less likely to enter a spontaneous remission period. 38
- Finally, due to the traumatic nature of this pathology, after its treatment and healing, it is possible that the patient may experience TMJC again if there are changes in habits, postures, or teeth, which could lead to a new impact and compression in the TMJC area.

## Conclusion

The present study shows the high response rate and safety of treatment for TMJC in patients with migraine and other ENT symptoms. In view of the results, new studies with larger sample sizes are recommended, and it is proposed that the scientific community introduces this new possibility of diagnosis and treatment for migraine and other otoneurological symptoms.
